# Manganese oxidation counteracts the deleterious effect of low temperatures on biofilm formation in *Pseudomonas* sp. MOB-449

**DOI:** 10.3389/fmolb.2022.1015582

**Published:** 2022-10-21

**Authors:** Lucila Ciancio Casalini, Ainelen Piazza, Fiorella Masotti, Betiana S. Garavaglia, Jorgelina Ottado, Natalia Gottig

**Affiliations:** Instituto de Biología Molecular y Celular de Rosario (IBR), Consejo Nacional de Investigaciones Científicas y Técnicas (CONICET) y Universidad Nacional de Rosario (UNR), Rosario, Argentina

**Keywords:** manganese, oxidation, groundwater, bioremediation, biofilm, *Pseudomonas*

## Abstract

Mn removal from groundwater by biological sand filter technology is negatively impacted by low temperatures in winter periods. Therefore, the need to study Mn(II)-oxidizing bacteria (MOB) having the potential to oxidize Mn(II) and form biofilms at low temperatures is imperative. These MOB can have potential as inocula for sand filter bioaugmentation strategies to optimize Mn removal during winter periods. We previously showed that a *Pseudomonas* sp. MOB-449 (MOB-449), isolated from a Mn biofilter, oxidizes Mn(II) in a biofilm-dependent way at low temperatures. In this work, MOB-449 Mn(II) oxidation and growth capacities were evaluated under planktonic and biofilm conditions at different temperatures. At 18°C, MOB-449 showed enhanced biofilm formation due to the addition of Mn(II) to the medium correlating with Mn(II) oxidation, compared to biofilms grown in control medium. Moreover, this enhancement on biofilm formation due to the addition of Mn(II) was only observed at 18°C. At this temperature, Mn(II) oxidation in membrane fractions collected from biofilms was induced by uncoupling oxidative phosphorylation from the electron transport chain with 2,4-Dinitrophenol. In *Pseudomonas*, a role for c-type cytochrome in Mn(II) oxidation has been demonstrated. Accordingly, transcriptional profiles of all terminal oxidases genes found in MOB-449 showed an induction of cytochrome c terminal oxidases expression mediated by Mn(II) oxidation at 18°C. Finally, heme peroxidase activity assays and MS analysis revealed that PetC, a cytochrome c5, and also CcmE, involved in the cytochrome c biogenesis machinery, are induced at 18°C only in the presence of Mn(II). These results present evidence supporting that cytochromes c and also the cytochrome c terminal oxidases are activated at low temperatures in the presence of Mn(II). Overall, this work demonstrate that in MOB-449 Mn(II) oxidation is activated at low temperatures to gain energy, suggesting that this process is important for survival under adverse environmental conditions and contributing to the understanding of the physiological role of bacterial Mn(II) oxidation.

## Introduction

Worldwide groundwater is an increasingly important source of safe water for drinking, agriculture and industry. As the presence of metals in groundwater is a growing concern, the need to find technologies that enable their removal is socially and environmentally relevant (Chen et al., 2019). Manganese as Mn(II), is a metal frequently found in groundwater that changes aesthetic and organoleptic water quality; interferes with its disinfection process; causes problems in the water distribution systems (Sly et al., 1990; Pacini et al., 2005); and affects human health (Whelton et al., 2007).

Biological sand filter technology, based on bacterial oxidation of metals to form insoluble oxides that can be filtered out of the water through sand filters, is widely used for groundwater potabilization since it is a cost-effective and eco-friendly technology that abolishes the addition of chemicals. This biological process is highly impaired by low temperatures in winter periods, mainly since bacterial growth and metabolic activities rates are reduced. Therefore, start-up periods for Mn removal are longer than at warmer temperatures (Cai et al., 2016; Ciancio Casalini et al., 2020).

Bioaugmentation of sand filters using bacteria with high Mn(II)-oxidizing and biofilm-forming capabilities provides an inexpensive, simple and efficient strategy for immobilizing these bacteria in sand filters and optimizing Mn removal (Bai et al., 2011; Li et al., 2016; McKee et al., 2016; Breda et al., 2018). Therefore, our previous work was focused on the isolation and characterization of environmental MOB with high Mn(II)-oxidizing and biofilm-forming capacities at different growth temperatures (Piazza et al., 2019), to select suitable candidates for bioaugmentation of biofilters (Ciancio Casalini et al., 2020). For one of the strains obtained, *Pseudomonas* sp. MOB-449 (MOB-449), Mn(II) oxidation has proven to be biofilm- and low temperatures-dependent (Piazza et al., 2019). We hypothesize that in this strain Mn(II) oxidation represent an adaptation to the biofilm lifestyle to overcome the detrimental effect of low temperatures.

While Mn(II) oxidation occurs in a diverse array of bacteria, and in various environmental niches (Stein et al., 2001; Tebo et al., 2010), the physiological function of bacterial Mn(II) oxidation in development or adaptation to the environment remains unclear (Geszvain et al., 2012). This process begins at the stationary phase when Mn(II)-oxidizing proteins catalyze the Mn(II) oxidation on the cell surface, forming a layer of insoluble Mn oxides (MnOx) (Tebo et al., 2010). Therefore, cells become covered with MnOx that prevent predation or viral attack (Geszvain et al., 2012), protect cells from radiation (Daly et al., 2007), and enable the oxidative degradation of natural organic matter or toxic compounds (Blöthe et al., 2015). Also, MOB exhibit increased resistance to oxidative stress (Banh et al., 2013). Recent *in vitro* studies have shown that Mn(II) oxidation is an exergonic reaction that occurs above pH 4 and uses various electron acceptors, including O_2_, and may provide energy to microorganisms (LaRowe et al., 2021). Additionally, it has been shown that *Candidatus Manganitrophus noduliformans* is able to couple extracellular Mn(II) oxidation to aerobic energy conservation (Yu and Leadbetter, 2020). Results in *P. putida* GB-1 and *P. putida* MnB1 showed that c-type cytochrome biogenesis-defective mutants loss Mn(II) oxidizing capability, suggesting that *Pseudomonas* also could acquire energy from Mn(II) oxidation, however this process has not yet been elucidated (Caspi et al., 1998; de Vrind et al., 1998).

Here, the impact of low temperatures and Mn(II) oxidation process in MOB-449 was studied revealing for the first time that Mn(II) oxidation is activated in biofilms to gain energy as survival strategy to cope with adverse environmental conditions. Overall, this work contributes to the understanding of the physiological role of bacterial Mn(II) oxidation.

## Materials and methods

### Strains, plasmids and growth conditions

The environmental MOB-449, isolated from a biological groundwater filter system (Piazza et al., 2019), was used in this study. For biofilm studies, MOB-449 was modified to express the green fluorescent protein (GFP). The *gfp*-expressing pMP2424 plasmid (Stuurman et al., 2000) was introduced by electroporation (Choi et al., 2006) into MOB-449, rendering MOB-449-pMP2424. Strains were grown at 30°C or 18°C in either lysogeny broth (LB) or in Lept media, without or with supplementation of 100 µM MnCl_2_ (Lept or Lept + Mn, respectively) (Boogerd and de Vrind, 1987). For MOB-449-pMP2424, gentamicin (Gm) 10 μg/ml was used.

### Quantification of bacterial growth and Mn(II) oxidizing capacities

To analyze bacterial growth, MOB-449 strain was grown in LB medium until an optical density at 600 nm (OD_600_) of 2.5. Then, cells were harvested by centrifugation at 5,000 rpm for 5 min, washed and the pellet was resuspended in the same volume of Lept medium. Aliquots of 0.1 ml culture were transferred to 100 ml of glass bottles containing 10 ml of Lept or Lept + Mn media and were incubated at 18°C or 30°C, with shaking at 200 rpm (planktonic growth) or statically (biofilm growth). At different time-points, planktonic growth was followed by measuring OD_600_ using a Synergy 2 Reader, BioTek. For biofilms, the supernatants were removed, and the biofilms were washed and resuspended in Lept medium by gently vortexing until complete resuspension. Serial dilutions of these biofilm suspensions were plated onto Lept-agar plates and bacterial growth was monitored by determination of Colony Forming Unit (CFU) ml^−1^.

To analyze Mn(II) oxidizing capacity of MOB-449 strain under different growth conditions, at the different time-points the MnOx present in biofilm suspensions or planktonic culture samples were quantified as previously described with the leucoberbelin blue reagent (LBB) (Piazza et al., 2019), and standardized to the amount of total proteins present in the samples quantified by the Bradford method (Bradford, 1976).

### Biofilm analysis by confocal laser scanning microscopy

Biofilms of the MOB-449-pMP2424 strain were set-up in Lept and Lept + Mn media, in 8-well microplates, as described above. The microplates were statically incubated at 18°C or 30°C and biofilms development was monitored daily by confocal laser scanning microscopy for 4 days. The GFP was excited at 488 nm and images were taken with LSM 880 Zeiss microscope. For each growth condition evaluated, 39 Z-stack images of three replicate wells were taken at distances of 0.7 µm and with a magnitude of ×20. The biofilms images are sample volumes of approximately 30 μm (height) × 140 μm (length) × 140 μm (width). To quantify the biofilm thickness and local biofilm density distribution, the biofilm image stacks were processed in BiofilmQ (https://drescherlab.org/data/biofilmQ/) as previously described (Hartmann et al., 2021), with minor modifications. First, noise fluorescence was removed by local averaging, that is Tophat-filtering. Second, the biofilm structure was binarized by thresholding using Otsu’s method. Third, the images were segmented with a cube side length of 1.8 µm. All cubes above the threshold length were considered for further investigation. Analysis of architecture local densities and thickness of biofilm parameter calculation after segmentation were obtained with the BiofilmQ analysis module. Visualization was performed using the 3D Heatmap/Kymograph tool in BiofilmQ. Biofilm analysis are representative of three biological replicates.

### Mn(II) oxidase activity quantification

Mn(II) oxidase activity of crude protein extracts from of MOB-449 strain grown under different conditions was evaluated by quantifying the conversion reaction of Mn(II) to MnOx *in vitro*. At different time-points, biofilms were collected, washed once with buffer 10 mM HEPES (pH = 7.5), and resuspended in 1 ml of the same buffer containing 1 mM phenylmethylsulfonyl fluoride (PMSF), sonicated on ice with a 3 mm probe with six cycles of 10 s ON and 1 min OFF with 25% amplitude (Sonics Materials TM VC 750 Ultrasonic Processor) and total proteins were quantified by Bradford method (Bradford, 1976). The *in vitro* Mn(II) oxidase activities were calculated from a reaction mixture, consisting of 0.020 mg ml^−1^ total protein extracts and 5 mM MnCl_2_ in 10 mM HEPES (pH = 7.5), incubated statically at 30°C for 3 h, quantifying the MnOx formed by the LBB method (Piazza et al., 2019). As control, to analyze if the appearance of MnOx was due to protein activity, mixtures were heat-treated (95°C for 15 min). Values were normalized by the initial activity, measured for each reaction mixture before the incubation at 30°C. Furthermore, differences in Mn(II)-oxidizing activity due to the presence of 2,4-dinitrophenol (DNP) were analyzed. To this end, membrane extracts were prepared as previously described (Ehrich and Salerno, 1990) and the reaction mixtures were supplemented with 5, 10 and 50 µM of DNP.

### Bacterial genome sequencing and bioinformatics techniques

MOB-449 genomic DNA was sequenced using Illumina technology platform (Illumina Inc. United States) as previously described (Masotti et al., 2021). The assembled genome of MOB-449 was deposited at DDBJ/ENA/GenBank under the accession JAMXMA000000000. The bacterial specie of MOB-449 could not be determined, but MIGA (http://microbial-genomes.org/) analyses demonstrated that its closed homolog is *Pseudomonas resinovorans* (88% Identity).

### RNA preparation and quantitative real-time PCR

RNA was extracted from 3-days-old biofilms suspensions grown at 18°C or 30°C in Lept or Lept + Mn media using 1 ml TRIzol reagent (Invitrogen) according to the manufacturer’s instructions. RNA was subjected to DNAse (Promega) treatment and cDNA was synthesized using M-MLV RT (Promega, United States). Gene specific primers used in this work are detailed in Supplemenatry Table S1. Then, RT-qPCRs were performed in a Mastercycler realplex thermal cycler (Eppendorf) using Platinum Taq DNA polymerase (Invitrogen) and SYBR Green I (Roche) to monitor double-stranded DNA (dsDNA) synthesis. The *15S* gene was used as internal reference control (Geszvain et al., 2013), values were normalized as previously described (Livak and Schmittgen, 2001).

### *In-gel* peroxidase assay for cytochrome c detection

Peroxidase activity of cytochrome c on sodium dodecyl sulfate polyacrylamide gels (SDS-PAGE) was detected as previously described (Thomas et al., 1976). Briefly, protein extracts were prepared from 3-days-old biofilms suspensions grown at 18°C or 30°C in Lept or Lept + Mn, as described in the Mn(II) oxidase activity section. Protein samples (150 µg) were prepared for electrophoresis by overnight dialysis at 4°C against 10 mM Tris-HCl, 1 mM EDTA, 20% glycerol and 5 pg ml^−1^ of pyronin Y and were run on an SDS-PAGE 15% with some changes. Sulfhydryl reducing agents were specifically omitted from the samples. The SDS concentration was 0.1% both in the electrophoresis buffer and the gels. The gels were pre-electrophoresed overnight at 1 mA per gel. The SDS was omitted during gel polymerization and was introduced into the gel by the pre-electrophoresis step. Electrophoresis was performed on gels at 4°C. The current was 1 mA per gel until the sample had completely entered the gel (∼1 h). The current was then increased to 3 mA per gel.

Peroxidase activity of cytochrome c was revealed by staining with a fresh solution of 6.3 mM 3,3′,5,5′-tetramethylbenzidine (TMBZ) in methanol. Immediately before use, 3 parts of the TMBZ solution were mixed with 7 parts of 0.25 M sodium acetate, pH = 5. The gel was immersed in the mixture at room temperature and protected from light with shaking every 10–15 min. After 2 h, H_2_O_2_ was added to a final concentration of 30 mM. Staining was visible within 3 min and increased by 30 min. At that time, the gel was placed in a solution of isopropanol: 0.25 M sodium acetate, pH = 5, in a 3:7 ratio. This solution was replaced twice with fresh solution to remove precipitated TMBZ (Thomas et al., 1976). For Coomassie brilliant blue staining, SDS-PAGE 15% with 15 µg of total proteins were run. Gels were scanned and ImageJ was used to quantify the intensity of bands. Differential bands were cut-out of the activity gels and mass spectrometry (MS) analyses were performed at the Mass Spectrometry Unit of the Institute of Molecular and Cellular Biology of Rosario (UEM-IBR), Argentina.

### Statistical analysis

Quantifications were performed from three biological replicates and three technical replicates per sample. All data were statistically analyzed using one-way ANOVA (*p* < 0.05) or by Student’s t-test (as indicated in Figure legends).

## Results

### Mn(II) oxidation only occurs in MOB-449 biofilms grown at low temperature

**FIGURE 1:**
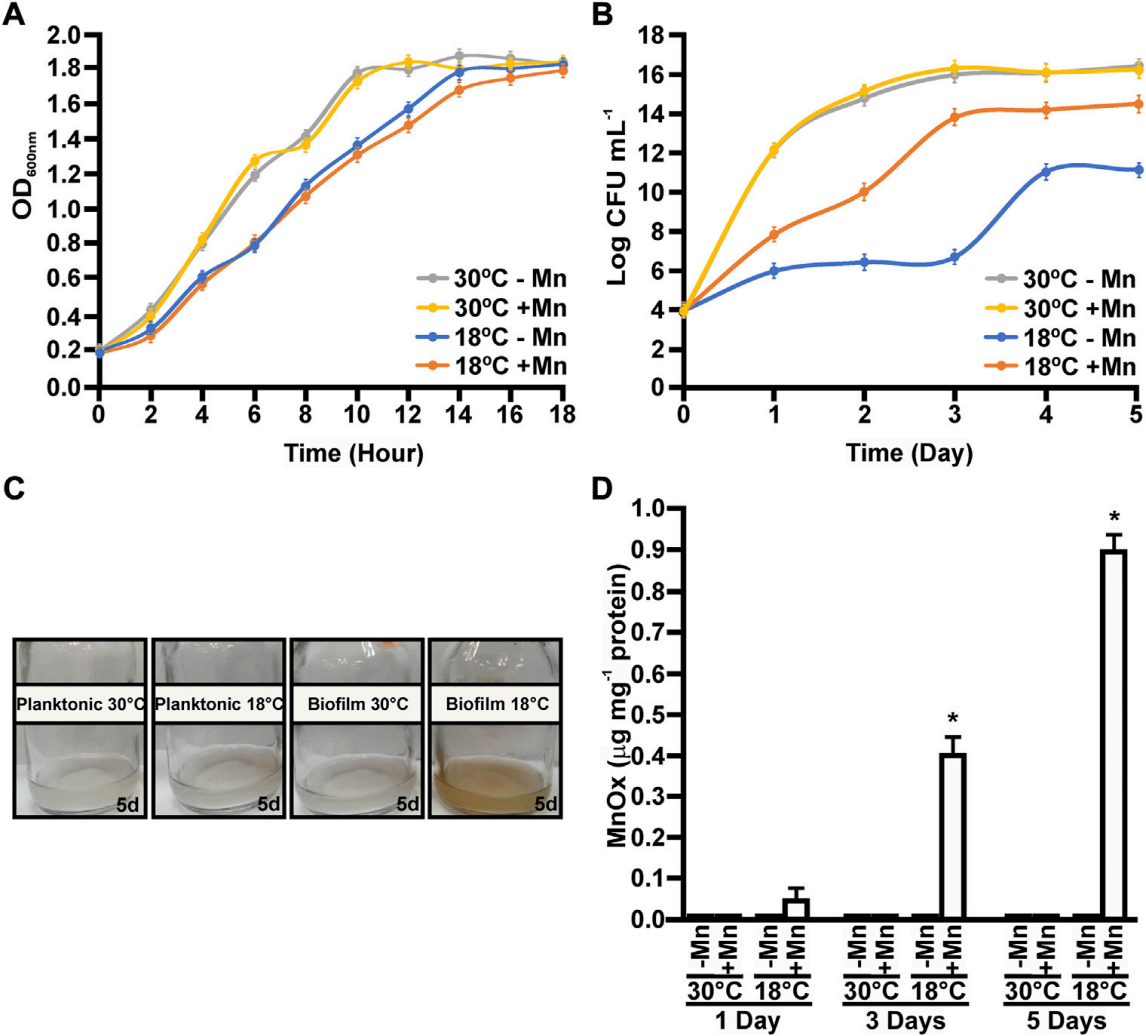
Growth and Mn(II)-oxidizing capacities of MOB-449 strain at different temperatures. MOB-449 was grown under planktonic measured as OD_600nm_ **(A)** or biofilm **(B)** as CFU mL^−1^ in Lept or Lept + Mn media at 18°C or 30°C. **(C)** Representative photographs of MOB-449 cultures. **(D)** Quantification of MnOx produced by MOB-449 expressed as μg of MnOx per mg of bacterial proteins under biofilm conditions using the LBB assay. All values represent the mean of three biological replicates and three technical replicates at each time point analyzed. Error bars indicate the SD. Data were statistically analyzed using one-way ANOVA and asterisks indicate significant differences (*p* < 0.05) between MnOx formed in Lept + Mn compared to Lept.

MOB-449 growth was analyzed under planktonic and biofilm conditions, in liquid Lept or Lept + Mn media at 18°C and 30°C. While low temperatures have a modest delay on bacterial growth, cells reached the same maximum OD_600_ value at both temperatures under planktonic growth conditions (Figure 1A). Moreover, no significant changes in growth were observed when Mn(II) was added into the medium (*p* < 0.05) (Figure 1A). Biofilm formation at 18°C was impaired when Mn(II) was not added to the medium, rendering slower growth and also a decrease in the final number of viable cells compared to the presence of Mn(II) that reverted this deleterious effect on biofilm viability (Figure 1B). Curiously, biofilm viability at 30°C was similar regardless the addition of Mn(II) (*p* < 0.05) (Figure 1B).

Mn(II) oxidation capacities were analyzed over the time for cultures grown at both temperatures. The production of MnOx was biofilm- and low temperature-dependent (Figures 1C,D). Under planktonic conditions, Mn(II) oxidation was impaired and no MnOx were detected at all assayed times (data not shown). In biofilms grown at 30°C, no Mn(II) oxidation was detected (Figures 1C,D). However, in biofilms grown at 18°C, MnOx were detected after 1 day of growth, evidenced by a characteristic brown color in the cultures (Figure 1C). Overall, these results showed that Mn(II) oxidation enhances biofilm growth at 18°C, compared to biofilms grown in Lept medium. Moreover, this enhancement on biofilm formation due to the addition of Mn(II) was only observed at 18°C. (Figures 1B,D).

### Mn(II) oxidation promotes MOB-449 biofilm formation at low temperatures

**FIGURE 2:**
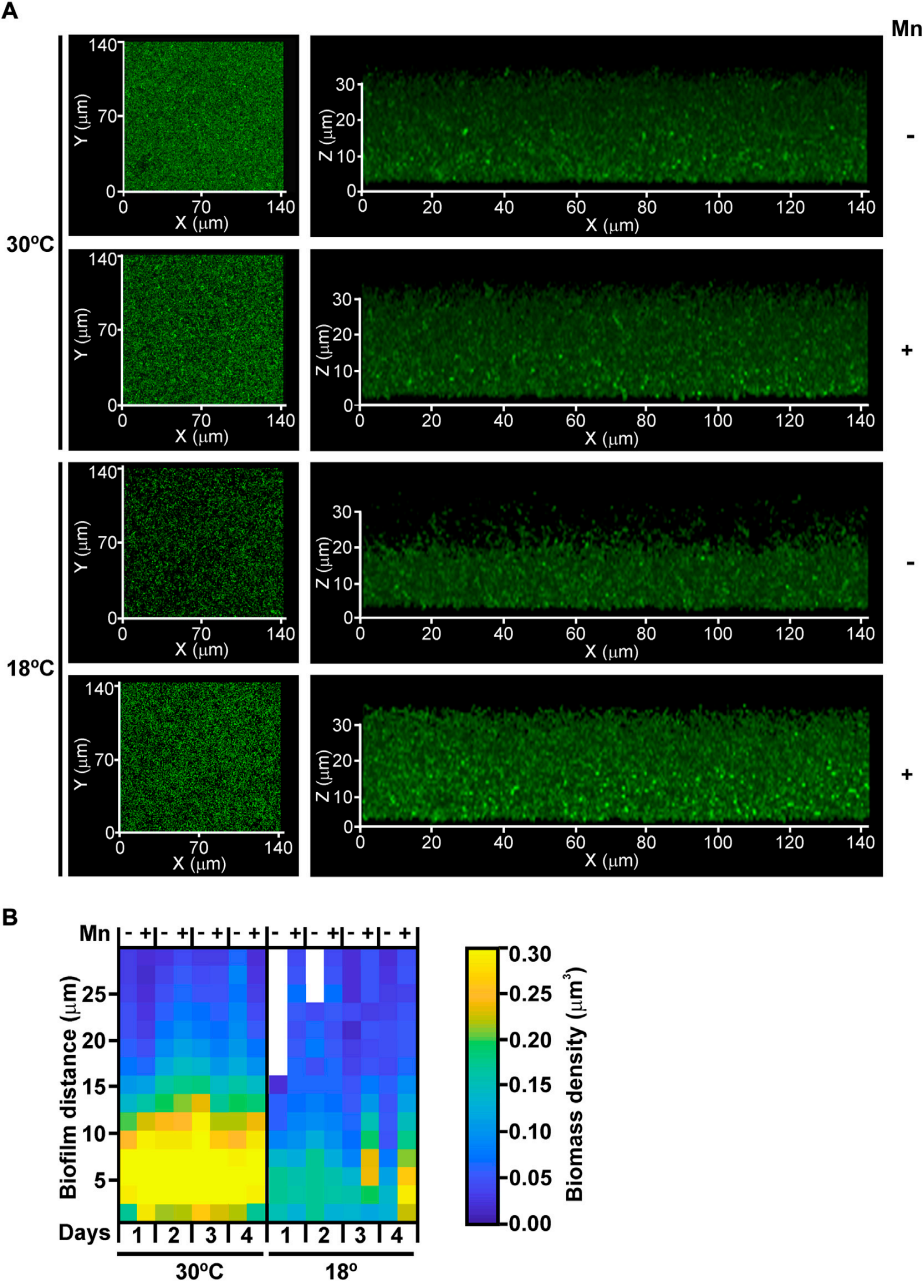
The effect of Mn(II) and temperature on biofilm development. **(A)** MOB-449-pMP2424 biofilms grown in Lept or Lept + Mn media at 18°C and 30°C were analyzed by confocal microscopy. Photographs of the xy and xz projections of three-dimensional MOB-449 biofilms acquired from 3-days-old biofilms are shown. The three-dimensional projections of 39 stacks were generated in Zeiss Black (Zeiss). Biofilm analysis are representative of three biological replicates. **(B)** Quantification of the biofilm biomass (colors intensity) during 4 days of growth in Lept or Lept + Mn media at 18°C and 30°C. The spatial distribution of the structural parameter -local density- for each condition is shown in the heatmap, the lack of color indicate the absence of biofilm at this depth. The 3D internal biofilm architectures are representative of three biological replicates.

The impact of Mn(II) and temperature on biofilm development was analyzed in more detail by confocal microscopy in MOB-449-pMP2424 grown in Lept or Lept + Mn media at 18°C and 30°C under static conditions. According to previous results (Figure 1), in Lept medium low temperatures had a negative effect on biofilm development as denoted by a decrease in both the biomass and thickness of the biofilm related to the one formed at 30°C (Figure 2A). However, this defect in biofilm development was attenuated when Mn(II) was added to the culture medium. At 30°C, no significant changes in biofilm growth were detected in Lept relative to Lept + Mn (Figure 2A).

Furthermore, quantification of biofilm thickness and local biomass density was performed. As evidenced in the heatmap, in Lept medium a better biofilm development was observed at 30°C than at 18°C (Figure 2B). However, at 18°C, both thickness and local biomass intensity were enhanced in Lept + Mn corroborating the positive effect of Mn(II) on biofilm formation at lower temperatures. As observed in biofilm growth assays (Figure 1B), the heatmap showed that at 30°C the addition of Mn(II) did not improve biofilm development (Figure 2B).

### Mn(II) oxidase activity is detected only in biofilms grown at low temperatures in the presence of Mn(II) and is enhanced by an uncoupling agent

**FIGURE 3:**
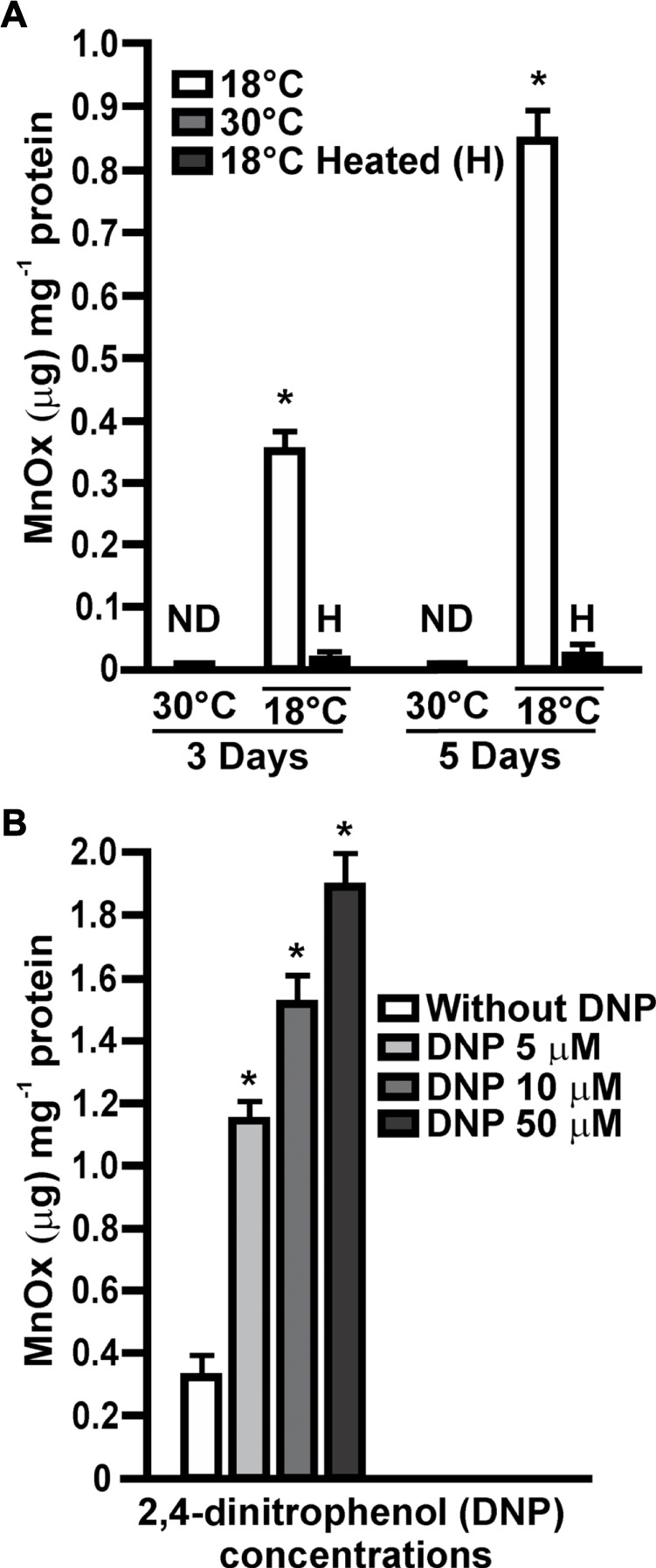
*In vitro* Mn(II) oxidase activities. **(A)** Mn(II) oxidase activities were assayed in total protein extracts obtained from MOB-449 biofilms grown in Lept + Mn for 3 and 5 days at 18°C or 30°C and expressed as μg of MnOx produced per mg of bacterial proteins. H indicates heat-treated samples. Quantifications were performed from three biological replicates and three technical replicates per sample. Mean values and SD are presented. Data were statistically analyzed using one-way ANOVA and asterisks indicate significant differences (*p* < 0.05) compared to the activity at 30°C **(B)** The same assays were performed with membrane extracts from 3-days-old MOB-449 biofilms grown at 18°C in Lept + Mn and the reactions were supplemented with different amount of DNP. Quantifications were performed from three biological replicates and three technical replicates per sample. Mean values and SD are presented. Data were statistically analyzed using one-way ANOVA and asterisks indicate significant differences (*p* < 0.05) compared to the control without DNP. ND indicates that no activity was detected.

Previous results showed that multiple biotic pathways and abiotic processes induce the oxidation of Mn(II) to MnOx (Geszvain et al., 2012). To determine if Mn(II) oxidation in MOB-449 is driven by a biotic pathway, *in vitro* Mn(II)-oxidase activity assays were performed with total proteins extracted from biofilms grown at different times at 18°C or 30°C in Lept + Mn medium. These assays revealed the presence of Mn(II) oxidase activity in biofilms grown at 18°C with a significant increment over the time (*p* < 0.05) (Figure 3A). However, no Mn(II)-oxidase activity was observed at 30°C (Figure 3A). These results are in agreement with the phenotypes of Mn(II) oxidation (Figure 1C) and with the quantifications of MnOx production (Figure 1D). *In vitro* Mn(II) oxidation was strongly impaired by the heat treatment (*p* < 0.05), consistent with the participation of proteins in the reaction. There was no evidence of Mn(II)-oxidase activity when biofilms were grown in Lept medium used as control (data not shown). These results suggest the increased production of proteins responsible for oxidizing Mn(II) at temperature which in turn cause an increase on the Mn(II)-oxidizing capacity of the bacteria.

The agent 2,4-dinitrophenol (DNP) is known to uncouple ATP synthesis, thereby permitting faster electron transport from the donor to a final acceptor (Harold, 1972). Assuming that Mn(II) is donating electrons to the transport chain to gain energy through a chemiosmotic mechanism, DNP may stimulate the Mn(II) oxidation process (Ehrich and Salerno, 1990). To analyze this hypothesis, Mn(II) oxidase activity assays were performed with membrane extracts obtained from 3-days-old biofilms grown at 18°C in Lept + Mn. In the presence of increasing concentrations of DNP a stimulation of Mn(II) oxidation reaction was observed (Figure 3B), suggesting that Mn(II) oxidation involves a chemiosmotic mechanism in MOB-449 biofilms grown at low temperatures.

### MOB-449 genome properties

MOB-449 genome was sequenced, and results revealed a 7,205,762 bp genome with a GC-content of 59.4% and 5,598 coding sequences. RAST annotation allowed the classification in subsystems. The most abundant categories are related to maintain basal cell functions such as: Amino Acids and derivatives metabolism, Carbohydrates metabolism, Protein metabolism, RNA Metabolism and Aromatic compounds metabolism that is a characteristic prominent metabolism in environmental *Pseudomonas*; Cofactors, Vitamins, Prosthetic Groups, Pigments; Membrane transport; Cell wall and capsule; Motility and chemotaxis, and Stress response (Supplementary Figure S1).

The presence of genes of the electron transport chain encoding terminal oxidases proteins, that catalyze reduction of molecular oxygen, was analyzed in the MOB-449 genome, by searching homologous sequences to those genes previously characterized in *P. aeruginosa.* In this bacterium, five terminal oxidases for aerobic respiration have been identified: three of them, the cbb3-1 oxidase (Cbb3-1), the cbb3-2 oxidase (Cbb3-2), and the aa3 oxidase (Aa3), are cytochrome c oxidases and the other two, the bo3 oxidase (Cyo) and the cyanide-insensitive oxidase (CIO), are quinol oxidases (Arai, 2011). The MOB-449 genome denoted the presence of five terminal oxidases with high-sequence homology to the corresponding genes in *P. aeruginosa* PAO1. Four of them are cytochrome c terminal oxidases: Cbb3-1, Cbb3-2 oxidase, Aa3 and a duplicate of the Aa3 oxidase named alternative-Aa3 oxidase that is not present in *P. aeruginosa* genome (Table 1). The other one is the cyanide-insensitive oxidase (CIO), a quinol oxidase (Table 1), and no homologous sequenced were found for the bo3 oxidase Cyo quinol oxidase. Accordingly, a similar distribution of the MOB-449 terminal oxidases proteins was found in the reference *P. resinovorans* NBRC 106553 strain (data not shown).

**TABLE 1:** List of genes in strain MOB-449 with homology to terminal oxidases from *P. aeruginosa* PAO1.

Gene	Locus tag in MOB-449	Predicted function	%Identity	%Similarity	E value
*ccoN1*	MOB-449_2142	cbb3-type cytochrome c oxidase subunit	89	95	0.00
*ccoO1*	MOB-449_2143	cbb3-type cytochrome c oxidase subunit	87	94	e^−138^
*ccoQ1*	MOB-449_2144	cbb3-type cytochrome c oxidase subunit	58	76	e^−21^
*ccoP1*	MOB-449_2145	cbb3-type cytochrome c oxidase subunit	72	87	e^−178^
*ccoN2*	MOB-449_2146	cbb3-type cytochrome c oxidase subunit	90	95	0.00
*ccoO2*	MOB-449_2147	cbb3-type cytochrome c oxidase subunit	93	97	e^−146^
*ccoQ2*	MOB-449_2148	cbb3-type cytochrome c oxidase subunit	67	82	e^−27^
*ccoP2*	MOB-449_2149	cbb3-type cytochrome c oxidase subunit	75	86	0.00
*coxA*	MOB-449_1177	aa3-type cytochrome c oxidase polypeptide I	94	97	0.00
*coxB*	MOB-449_1176	aa3-type cytochrome c oxidase polypeptide II	82	91	0.00
*coxC*	MOB-449_1179	aa3-type cytochrome c oxidase polypeptide III	89	94	0.00
*coxM*	MOB-449_4930	alternative aa3-type cytochrome c oxidase polypeptide II	26	45	e^−6^
*coxN*	MOB-449_4929	alternative aa3-type cytochrome c oxidase polypeptide I	41	57	e^−127^
*coxP*	MOB-449_4931	alternative aa3-type cytochrome c oxidase polypeptide III	32	51	e^−20^
*cioA*	MOB-449_2005	cyanide-insensitive quinol oxidase subunit	86	91	0.00
*cioB*	MOB-449_2004	cyanide-insensitive quinol oxidase subunit	84	93	0.00

Like in *P. aeruginosa* PAO1 (Arai, 2011), in MOB-449, both cbb3-type cytochrome c oxidases are encoded by two tetracistronic operons *ccoNOQP1* and *ccoNOQP2* tandemly clustered (cluster 1: MOB-449_2142 to MOB-449_2145 and cluster 2: MOB-449_2146 to MOB-449_2149). Also, the three subunits that compose the aa3-type cytochrome c oxidase are encoded by *coxA* (MOB-449_1176), *coxB* (MOB-449_1177) and *coxC* (MOB-449_1179) genes. The alternative-aa3-type oxidase is encoded by *coxM* (MOB-449_4930), *coxN* (MOB-449_4929) and *coxP* (MOB-449_4931) genes. Finally, two genes encode the subunits of the CIO, *cioA* (MOB-449_2005) and *cioB* (MOB-449_2004) (Table 1).

### The expression of cytochrome c terminal oxidases is induced by Mn(II) in MOB-449 biofilms grown at low temperatures

**FIGURE 4:**
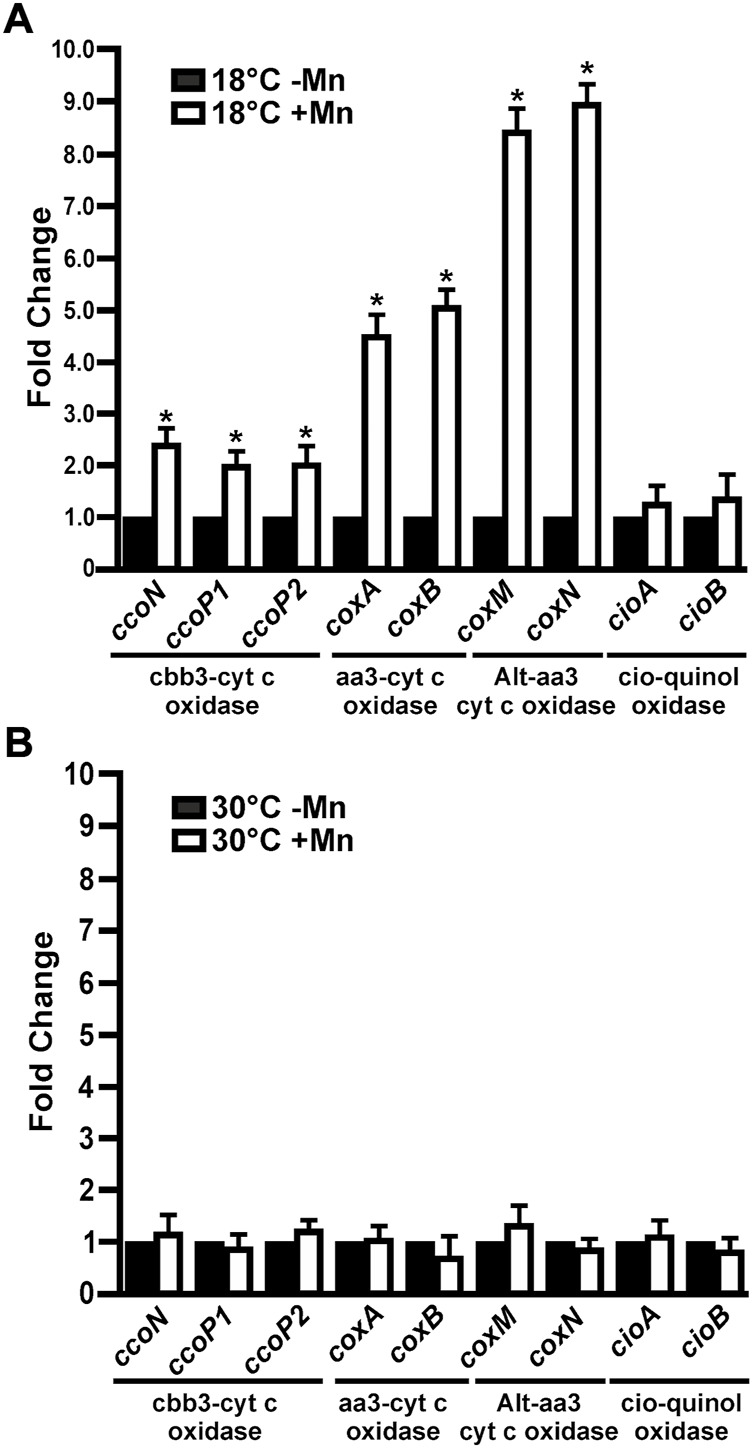
Expression of MOB-449 terminal oxidases genes under biofilm conditions. MOB-449 biofilms were grown in Lept or Lept + Mn at 18°C **(A)** or 30°C **(B)** and subjected to RT-qPCR assays to analyze the expression of *ccoN*, *ccoP1*, *ccoP2*, *coxA*, *coxB*, *coxM*, *coxN*, *cioA* and *cioB*. The *15S* gene was used as internal control for the calculation of relative gene expression. White bars indicate the expression levels of the genes in Lept + Mn relative to the expression levels in Lept (Black bars). Values are the means of three biological replicates with three technical replicates each. Error bars indicate standard deviation. Data were analyzed by Student’s t-test and asterisks indicate significant differences (*p* < 0.05) between samples grown with the addition or not of Mn(II).

Expression levels of the terminal oxidases in 3-days-old biofilms grown at 18°C or 30°C in Lept or Lept + Mn media were quantified by RT-qPCR (Figure 4). The corresponding genes of *ccoNOQP1* and *ccoNOQP2* operons have highly homologous sequences, except for *ccoP1* and *ccoP2*. Therefore the assays were performed with oligonucleotides that co-amplified both *ccoN1* and *ccoN2* and with specific oligonucleotides for each *ccoP1* and *ccoP2* (Supplementary Table S1). Specific oligonucleotides were designed also for *coxA* and *coxB* encoding the subunits of the Aa3 terminal oxidase, *coxM* and *coxN* genes of the alternative-Aa3 oxidase genes and for *cioA* and *cioB* genes of the CIO terminal oxidase (Supplementary Table S1). At 18°C, the genes encoding the four cytochrome c oxidases showed a significantly increased expression in the presence of Mn(II) relative to their expression when bacteria were grown with no Mn(II) added (*p* < 0.05) (Figure 4A). *ccoN*, *ccoP1* and *ccoP2*, showed increase in transcript levels ≈ 2 times (*p* < 0.05), whereas *coxA* and *coxB* showed a major induction of 4.5 and 5 times, respectively. The highest expression levels were detected for *coxM* and *coxN*, showing a fold change of 8.5 and 9 in the presence of Mn(II), respectively (*p* < 0.05). On the contrary, *cioA* and *cioB* genes encoding for the quinol oxidase showed no significant changes in the level of expression relative to the control Lept medium (*p* < 0.05) (Figure 4A). At 30°C, the expression of all assayed terminal oxidases genes was unaffected in the presence of Mn(II) (Figure 4B).

### MOB-449 biofilms cytochrome c transcription profiles suggest their role in channeling electrons from Mn(II) into the electron transfer chain

**FIGURE 5:**
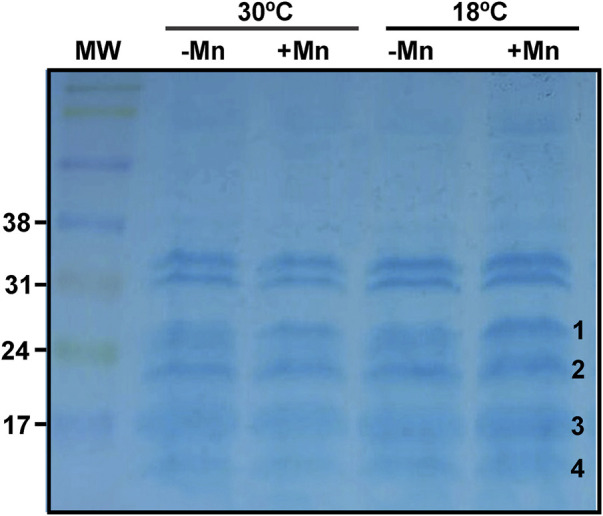
Profiles of the MOB-449 biofilm c-type cytochromes. Protein extracts of MOB-449 biofilms grown in Lept or Lept + Mn at 30°C or 18°C were analyzed by SDS-PAGE and the c-type cytochromes were revealed *via* their peroxidase activities using TMBZ staining. Four positive stained and differentially detected bands of proteins were cut-out of the activity gel (numbered at the right of the band) and identified by MS analysis.

In order to find c-type cytochromes possibly involved in Mn(II) oxidation, their presence in total protein extract of 3-days-old biofilms grown at 18°C or 30°C in Lept or Lept + Mn media were examined by *in-gel* peroxidase activity assays (Thomas et al., 1976). At least six bands with a well-defined TMBZ staining were found (Figure 5). These bands showed no changes in abundance when obtained from biofilms grown at 30°C with the addition or not of Mn(II). On the contrary, at 18°C, four bands showed an enhanced staining in the presence of Mn(II) (Figure 5). These four bands were cut-out of the gel and identified by MS analysis (Table 2). The highest band (MW ∼29 kDa) was identified as the cytochrome c1, named PetC. The band of 22–23 kDa revealed the presence of peptides of both CcoO1 and CcoO2. The ∼16 kDa corresponded to the cytochrome c-type biogenesis protein named CcmE and the ∼14 kDa to a cytochrome c5.

**TABLE 2:** Nano LC-MS/MS analysis of the bands extracted from TMBZ gels of 3-day-old MOB-449 biofilms grown at 18°C or 30°C in Lept or Lept + Mn media.

	Accession	Description
Protein band 1	MOB449_5366 (in MOB-449) S6AMN6 (in *P. resinovorans* NBRC 106553)	Cytochrome C1, Ubiquinol cytochrome C oxidoreductase subunit, PetC, MW = 29.10 kDa
	Sequence	Modifications
	GTDWLYSYLRGQPVFIVR	—
	LTEAEFDEK	—
Protein band 2	Accession	Description
	MOB449_2143 (in MOB-449) S6AGS6 (in *P. resinovorans* NBRC 106553)	Cbb3-type cytochrome c oxidase subunit CcoO1, MW = 22.67 kDa
	Sequence	Modifications
	AMQTLGVPYSDEDVAGAQAAVK	M1(Oxidation)
	Accession	Description
	MOB449_2147 (in MOB-449) S6AY10 (in *P. resinovorans* NBRC 106553)	Cbb3-type cytochrome c oxidase subunit CcoO2, MW = 22.72 kDa
	Sequence	Modifications
	GKTEMDALVAFLQGLGTSIK	M1(Oxidation)
	MKQHELVEK	M1(Oxidation)
	MPSYPWLVENK	M1(Oxidation)
	TALELEGR	—
	TEMDALVAFLQGLGTSIK	M1(Oxidation)
Protein band 3	Accession	Description
	MOB449_122 (in MOB-449) S6ALM5 (in *P. resinovorans* NBRC 106553)	Cytochrome c-type biogenesis protein CcmE, heme chaperone, MW = 15.97 kDa
	Sequence	Modifications
	YHGILPDLFR	—
Protein band 4	Accession	Description
	MOB449_3459 (in MOB-449) S6BQD1 (in *P. resinovorans* NBRC 106553)	Cytochrome c5, MW = 14.09 kDa
	Sequence	Modifications
	GTCADCTDDELK	M2(Oxidation)
	TPDDIIAK	—

## Discussion

Notwithstanding new information on the diversity of Mn(II)-oxidizing bacteria and the elucidation of molecular mechanisms likely to be involved in this process, factors controlling the physiological function of Mn(II) oxidation reaction in bacteria remain unclear (Stein et al., 2001; Tebo et al., 2010). In this work, we took a different approach to identify novel factors mediating Mn(II) oxidation in the MOB-449 strain, isolated from a biological sand filtration system. We previously characterized this strain and showed its ability to oxidize Mn(II) under biofilm conditions at low temperatures (Piazza et al., 2019). Here we expand these studies, to show a deleterious effect of low temperatures on MOB-449 biofilm development but not on planktonic growth. Interestingly, this defect in MOB-449 biofilms survival is attenuated by the presence of Mn(II) and concurrent with the oxidation of this metal. Moreover, conditions where MOB-449 growth was proficient showed impairment of Mn(II) oxidation. These results suggest an important point concerning possible influences of Mn(II) oxidation on MOB-449 biofilm development and a contribution to bacterial survival under adverse conditions.

Previously, we have performed bioaugmentation assays with another MOB, *Pseudomonas sagittaria* MOB-181. Differently to MOB-449, MOB-181 achieved the best Mn(II) oxidizing activity at 37°C, while it also can oxidize Mn(II) at 18°C but at lower efficiency than MOB-449 (Piazza et al., 2019). Bioaugmentation with MOB-181 optimizes Mn removal under summer (lowest average and highest average temperatures of influent water of 18°C and 29°C, respectively) and winter (lowest average and highest average temperatures of influent water of 7°C and 17°C, respectively) periods. However, in winter periods despite the presence of MOB-181, the start-up of Mn removal still requires additional optimization (Ciancio Casalini et al., 2020). Hence, the possibility to count with MOB-449 that can efficiently oxidize Mn(II) at temperatures around the maximal and minimal ones during the winter season, makes this strain an optimal candidate for optimization of Mn(II) removal process during cold months. Little is known regarding which physiological role Mn(II) oxidation plays in bacteria, only in a recent work it has been shown that bacteria are able to use the extracellular reaction of Mn(II) oxidation to obtain energy for growth (Yu and Leadbetter, 2020). In addition, it has been proposed that Mn(II) may act as an electron donor in the electron transport chain through cytochromes c (Caspi et al., 1998; de Vrind et al., 1998; Yu and Leadbetter, 2020). Our results show that DNP stimulates Mn(II) oxidation in membrane fractions extracted from MOB-449 biofilms grown at 18°C suggesting a coupling of energy acquirement with the oxidation process.

The electron transport chain has been extensively studied in the opportunistic pathogen *P. aeruginosa*. This bacterium has three cytochrome c terminal oxidases that receive electrons *via* c-type cytochromes: Cbb3-1, Cbb3-2 and Aa3; and two quinol oxidases that receive electrons directly from ubiquinol: Cyo and CIO (Arai, 2011). MOB-449 genome denotes the presence of the cytochrome c terminal oxidases Cbb3-1, Cbb3-2, Aa3 and Alternative-Aa3, and the quinol oxidase CIO. The upregulation in MOB-449 biofilms of the genes encoding the cytochrome c terminal oxidases at 18°C in the presence of Mn(II), suggest that the electron transport chain is activated. In addition, since cytochrome c terminal oxidases but not the quinol oxidase were induced, these results suggest a role for c-type cytochromes in channeling electrons from Mn(II) to cytochrome c terminal oxidases to improve energy production.

In *P. aeruginosa*, the differential use of terminal oxidases under different growth conditions contribute to the survival and ubiquity of this bacterium in various environmental niches (Arai, 2011). Aa3, and CIO have low affinity for oxygen, but Cbb3-1 and Cbb3-2 are high affinity enzymes. Therefore, Cbb3 oxidases are known to be induced under low oxygen conditions; Cbb3-1 plays a primary role in aerobic growth irrespective of oxygen concentration; and Cbb3-2 plays a compensatory or supplementary role under oxygen-depleted conditions (Comolli and Donohue, 2004; Alvarez-Ortega and Harwood, 2007; Kawakami et al., 2010). In *P. aeruginosa*, Aa3 has the highest efficiency to create a proton gradient across cell membranes, but it is not expressed under normal growth conditions. Aa3 has been shown to be utilized only under starvation conditions for efficient energy production and its utilization might be advantageous for proliferation in natural oligotrophic environments (Arai, 2011). *cioAB* genes are upregulated at stationary phase or at very low oxygen concentrations but specially in the presence of the respiratory chain inhibitors, cyanide and sodium nitroprusside (SNP) (Kawakami et al., 2010). In MOB-449, genes encoding Aa3 oxidases showed the highest expression levels. This transcriptional regulation suggest a link between Mn(II) oxidation and the action of these terminal oxidases, which are the most efficient to produce energy (Arai, 2011), under the adverse growth conditions. Moreover, because the alternative-Aa3 oxidase, that is absent in *P. aeruginosa,* was the most induced complex in MOB-449, it is possible that this second complex represents an example of bacterial adaptation to the environment.

Iron-oxidizing bacteria acquire energy from Fe(II) oxidation through an outer membrane c-type cytochrome (Cyc2 or Cyt572) used as the initial oxidant and carrier to the electron transport chain for the Fe(II)-derived electron (Castelle et al., 2008; Jeans et al., 2008). In these bacteria an alternative mechanism involves a porin–cytochrome c protein complex (PCC1-PCC3) (Richardson et al., 2012). *C. Manganitrophus noduliformans* use both mechanisms to deliver electrons from Mn(II) oxidation (Yu and Leadbetter, 2020). By homology searches we were unable to find genes encoding these characterized cytochrome c in the MOB-449 genome. Therefore, in order to identify cytochrome c proteins in MOB-449, heme peroxidase activity assays were performed to find proteins with enhanced activity at 18°C in the presence of Mn(II). An increased amount of PetC and cytochrome c5 was found at 18°C in the presence of Mn(II). These results suggest that both cytochromes are involved in the process of Mn(II) oxidation in MOB-449, although, the participation of additional cytochromes could not be ruled out. PetC is a cytochrome c1 (containing a covalently bound heme C) that along with the Rieske Fe-S protein (PetA) and the cytochrome b (PetB) constitute the ubiquinol-cytochrome c2 oxidoreductase (bc1 complex). The cytochrome bc1 complex is a bacterial respiratory chain central redox carrier. It receives electrons from the quinone/quinol pool in the membrane, delivering these electrons to reduce metalloprotein electron carriers, which are in most cases c-type cytochromes. Simultaneously, it produces vectorial proton translocation, leading to an electrochemical proton gradient across the membrane. Analysis of the probable localization of MOB-449 PetC by psortb (https://www.psort.org/psortb/) predicts periplasmic, outer membrane and extracellular location for this protein (Gardy et al., 2003). In MOB, MnOx are accumulated at the extracellular space (Geszvain et al., 2012), therefore, it is possible that PetC may be directly involved in obtaining additional electrons from Mn(II) oxidation reaction, and through the bc1 complex, deliver them to the transport chain to gain further energy for biofilm development. The cytochrome c5 found by the heme peroxidase activity assays showed a similar subcellular location than PetC and may also be a receptor of Mn(II) oxidation electrons, delivering them to the terminal cytochrome c oxidases.

In accordance to RT-qPCR assays that indicate a higher expression levels of the genes encoding Cbb3-1 and Cbb3-2 cytochrome c terminal oxidases at 18°C in the presence of Mn(II), heme peroxidase activity assays results showed a higher abundance of CcoO1 and CcoO2 under these growth conditions. CcoO1 and CcoO2 can be detected in the activity assay since both have a cytochrome c domain (Sandri et al., 2018), that was corroborated for both MOB-449 proteins by the prosite domain scan (https://prosite.expasy.org/) (Hulo et al., 2006). RT-qPCR also showed an increment of *cox* gene expression under this condition. MOB-449 CoxB and CoxM have a predicted cytochrome c domain (https://prosite.expasy.org/) similar to their bacterial homologs (Sandri et al., 2018). Further, bands with increased staining at 18°C in the presence of Mn(II) in the TMBZ/SDS-PAGE section corresponding to the MW of these proteins (40–60 kDa) were observed. However, these bands did not present enough activity and were not sufficiently defined to cut them out of the gel for further analysis.

CcmE, a periplasmic protein that is N-terminally anchored to the membrane and involved in cytochrome c biogenesis, was detected among the analyzed proteins. The protein has a strictly conserved histidine involved in heme binding and acts as a periplasmic heme carrier, preventing the aggregation of heme (Thöny-Meyer, 1997). As mentioned, previous results showed that *P. putida* MnB1 and GB-1 insertions mutants in different genes (*ccmF*, *ccmF*, *ccmA*, and *ccmE*) involved in cytochrome c biogenesis, resulted in the absence of cytochromes c with a concomitant loss of Mn(II) oxidizing activity (Caspi et al., 1998; de Vrind et al., 1998). Therefore, the enhanced CcmE production, and the presence of PetC and cytochrome c5 observed in this study, supports a role for cytochrome c in the Mn(II) oxidation process, contributing to Mn(II) oxidation by MOB-449 biofilms at low temperatures.

Different hypothesis have been proposed for the link between cytochromes c biogenesis and Mn(II) oxidation in *P. putida* strains MnB1 and GB-1. First, cytochrome c may be involved in electron transfer from Mn(II) to oxygen. Alternatively, cytochrome c may be part of an outer membrane Mn(II)-oxidizing complex. A third possibility is that either the products of the cytochrome c maturation genes or c-type cytochromes themselves are involved in the biosynthesis of other molecules which may play a role in Mn(II) oxidation (Caspi et al., 1998; de Vrind et al., 1998). The results of this work are in line with the first possibility, since we present evidence supporting the biogenesis of cytochrome c, two cytochromes c and the cytochrome c terminal oxidases, are activated in the presence of Mn(II). Furthermore, this activation correlates with Mn(II) oxidation and MOB-449 biofilm development, suggesting that Mn(II) oxidation is used to gain energy and survive under adverse environmental conditions.

Previous studies in iron-oxidizing *Zetaproteobacteria* and other neutrophilic Fe-oxidizers have shown that the membrane c-type cytochrome Cyc2 and cytochrome c terminal oxidases are highly expressed in Fe(II)-rich environments and their expression is also stimulated by Fe(II) addition to bacterial cultures (Jewell et al., 2016; McAllister et al., 2020; Zhou et al., 2022). These results, in agreement with our results in MOB-449, suggest a transcriptional regulation mediated by Fe(II) and Mn(II) to allow the electrons derived from their oxidation reactions to be passed through cytochrome c terminal oxidases to reduce oxygen and acquire energy for growth (Jewell et al., 2016; McAllister et al., 2020; Zhou et al., 2022). Particularly for MOB-449, this activation occurs in a detrimental bacterial growth condition such as biofilm exposed at low temperatures. However, the signaling and transcription factors implicated in this transcriptional regulation are unknown. Future studies will be needed to clarify how metals induce the bacterial machinery to gain energy from metal oxidation reactions.

## Conclusion

The results of this work demonstrated that in *Pseudomonas sp.*MOB-449, Mn(II) oxidation is activated to gain energy, revealing a new role for this process in biofilm development under detrimental environmental conditions, and contributes to the understanding of the physiological role of bacterial Mn(II) oxidation.

## Data Availability

The datasets presented in this study can be found in online repositories. The names of the repository/repositories and accession number(s) can be found in the article/Supplementary Material

## Supplementary material

Supplementary Figure S1 Subsystem classification of MOB-449 genome. MOB-449 genome was sequenced, and its properties revealed by RAST annotation (https://rast.nmpdr.org/) are shown.

Supplementary Figure S2 Loading control of the normalized protein levels in samples used for the peroxidase activity assays (Figure 5). Protein extracts of MOB-449 biofilms grown in Lept or Lept + Mn at 30°C or 18°C were quantified by the Bradford method (Bradford, 1976), then 15 µg of total proteins for each extract were analyzed by SDS-PAGE 15% and stained with Coomassie brilliant blue to verify that the same amount of proteins were loaded for peroxidase activity assays.
