# Supplementary material for: Manganese oxidation counteracts the deleterious effect of low temperatures on biofilm formation in Pseudomonas sp. MOB-449
Source: Front Mol Biosci. 2022 Oct 21;9:1015582. doi: 10.3389/fmolb.2022.1015582 (PMC9634551; doi:10.3389/fmolb.2022.1015582)
Supplement: Supplementary file 1 [file Table1.DOCX]

**Supplementary Material**

**Table S1**: Oligonucleotides used for qRT-PCR.

| **Oligonucleotide** | **Sequence (5´…3´)** |
| --- | --- |
| 15S-F  15S-R  CcoP1 -F  CcoP1 -R  CcoP2-F  CcoP2-R  CoxA-F  CoxA-R  CoxB-F  CoxB-R  CoxM-F  CoxM-R  CoxN-F  CoxN-R  CioA-F  CioA-R  CioB-F  CioB-R | CCGCCAACATCAACAAGC  TCTTTGCCCTTCAGGTAGTC  GGCTATGCCGATGGCTGGA  TAGACATCGCGGAGTAGCG  AGTGACCGAAGGCAAGCCG  GCGTACTTGGCGAAGATCG  GCACTACGGTGGAAATCC  GGAGGTGTCGTAGATGTCG  TGGAGACTGACATGAGTGCA  CTTGTGGTTGGTGGTCAGC  ATTTCTACATCCCGCAGATACG  AACTCCGCACAGAGGATTTC  CATTCGGCCCAGGAACTG  CTTGAAGGTGCTCCAGGC  ACCTGGCTGATCATGAAGAC  GGTCCAGATGCTGACGATAC  AAGGCAAGGAACAGGGTATC  TAGCGTTGTCGTCATCATCC |
