# Supplementary figures and images for: Manganese oxidation counteracts the deleterious effect of low temperatures on biofilm formation in Pseudomonas sp. MOB-449
Source: Front Mol Biosci. 2022 Oct 21;9:1015582. doi: 10.3389/fmolb.2022.1015582 (PMC9634551; doi:10.3389/fmolb.2022.1015582)

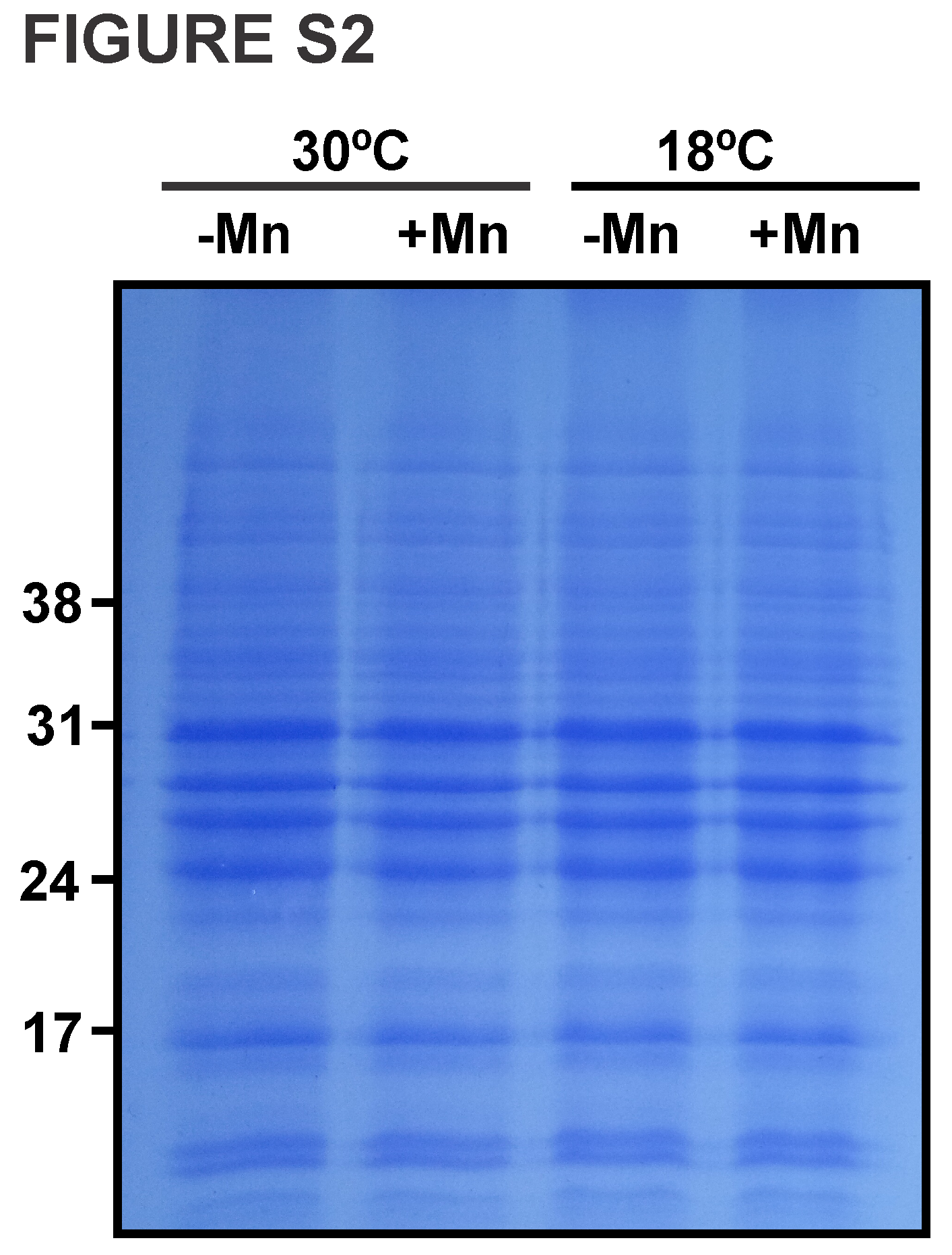

Supplement: Supplementary file 2 [file Image2.tif]

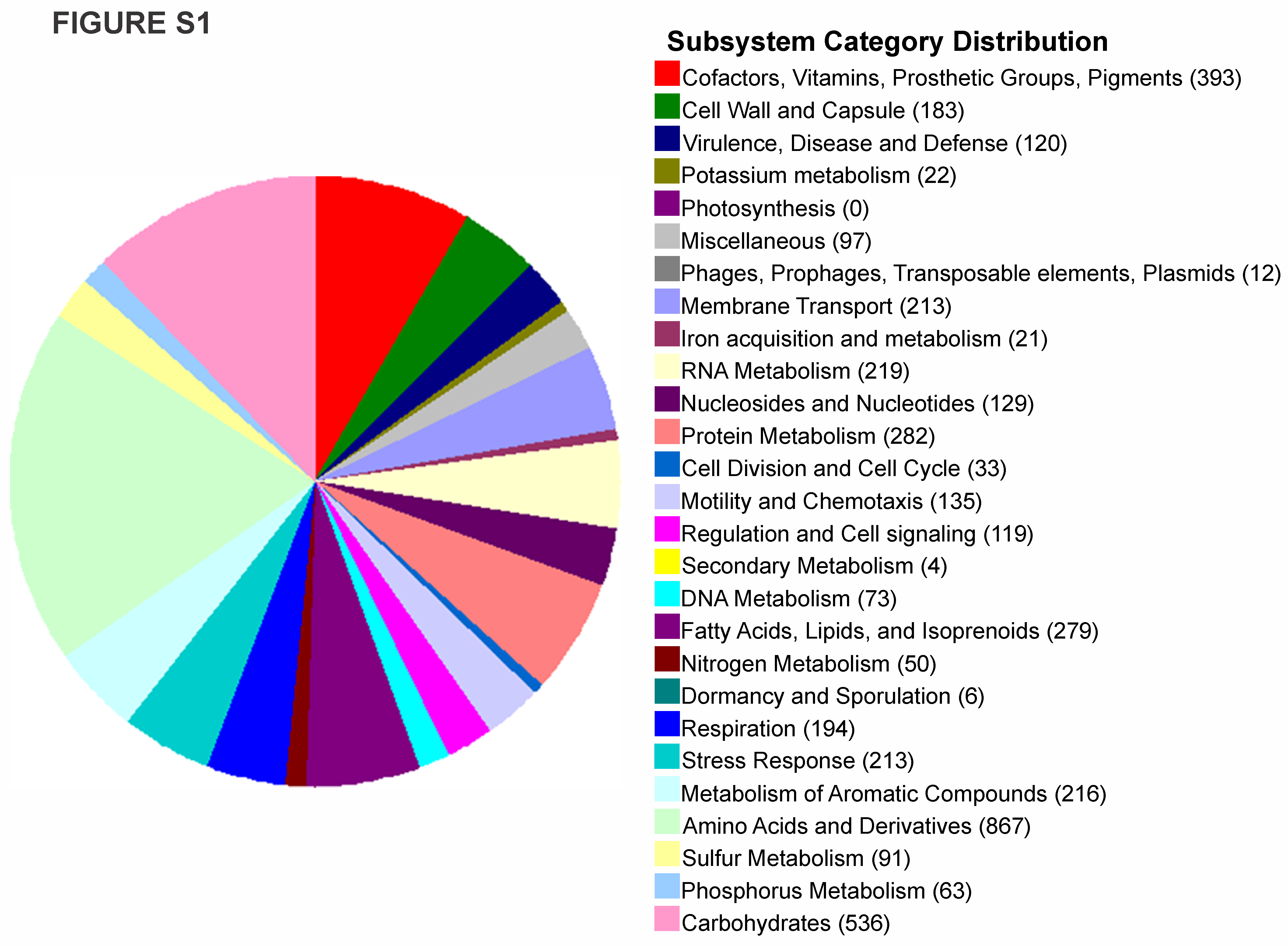

Supplement: Supplementary file 3 [file Image1.tif]
